# A HuGE Review and Meta-Analyses of Genetic Associations in New Onset Diabetes after Kidney Transplantation

**DOI:** 10.1371/journal.pone.0147323

**Published:** 2016-01-20

**Authors:** Katherine Angela Benson, Alexander Peter Maxwell, Amy Jayne McKnight

**Affiliations:** 1 Centre for Public Health, Queen's University Belfast, Belfast, United Kingdom; 2 Regional Nephrology Unit, Belfast City Hospital, Belfast, United Kingdom; University of Toledo, UNITED STATES

## Abstract

**Purpose:**

New onset diabetes after transplantation (NODAT) is a serious complication following solid organ transplantation. There is a genetic contribution to NODAT and we have conducted comprehensive meta-analysis of available genetic data in kidney transplant populations.

**Methods:**

Relevant articles investigating the association between genetic markers and NODAT were identified by searching PubMed, Web of Science and Google Scholar. SNPs described in a minimum of three studies were included for analysis using a random effects model. The association between identified variants and NODAT was calculated at the per-study level to generate overall significance values and effect sizes.

**Results:**

Searching the literature returned 4,147 citations. Within the 36 eligible articles identified, 18 genetic variants from 12 genes were considered for analysis. Of these, three were significantly associated with NODAT by meta-analysis at the 5% level of significance; *CDKAL1* rs10946398 p = 0.006 OR = 1.43, 95% CI = 1.11–1.85 (n = 696 individuals), *KCNQ1* rs2237892 p = 0.007 OR = 1.43, 95% CI = 1.10–1.86 (n = 1,270 individuals), and *TCF7L2* rs7903146 p = 0.01 OR = 1.41, 95% CI = 1.07–1.85 (n = 2,967 individuals).

**Conclusion:**

Evaluating cumulative evidence for SNPs associated with NODAT in kidney transplant recipients has revealed three SNPs associated with NODAT. An adequately powered, dense genome-wide association study will provide more information using a carefully defined NODAT phenotype.

## Introduction

New onset diabetes after transplantation (NODAT), also known as post transplantation diabetes mellitus (PTDM), is a serious complication of solid organ transplantation [1]. It affects 2–50%[1–3] of organ transplant recipients and is associated with greater healthcare costs and an increased risk of graft failure, cardiovascular complications and death [4]. The wide variation in reported prevalence of NODAT in part reflects the varying clinical definitions of this disorder. In different clinical studies the NODAT phenotype has been defined by various criteria including elevated fasting blood glucose; abnormal oral glucose tolerance tests; elevated glycated haemoglobin (HbA1c) or absolute requirement for hypoglycaemic therapies following solid organ transplantation [5,6]. A number of modifiable and non-modifiable risk factors have been identified which may predict NODAT. Modifiable risk factors include obesity and choice of anti-rejection immunosuppression medication [7]. Patients receiving tacrolimus-based immunosuppressive regimens are at greater risk of developing NODAT compared to those prescribed ciclosporin-based immunosuppressive treatment [8]. However, choosing an immunosuppressive regimen to specifically avoid NODAT may have a damaging effect on the graft itself [1]. Non-modifiable risk factors include family history of diabetes mellitus, polycystic kidney disease, hepatitis C infection, female gender and older recipient age [9,10]. There is an established genetic component to NODAT, however the identification of genetic risk factors has proved challenging. It is well documented that ethnicity is an important risk factor; people of African American, Hispanic, or South Asian background are at a significantly increased risk of developing the disease [5]. Low plasma adiponectin concentration, a factor which is under significant genetic control [11], has also been demonstrated to be predictive for NODAT [12]. Genome-wide association studies (GWAS) are revealing SNPs associated with diabetes, which are replicated across multiple populations [13,14], but such robust multi-centre GWAS have not yet been published for NODAT. However, multiple publications have reported genetic associations with NODAT in the literature, often with inconsistent results [1]; this report describes an inclusive review and meta-analysis of existing data.

## Materials and Methods

### Selection Criteria

Review of the literature was performed to identify all published genetic variants associated with NODAT in a kidney transplant population. Studies carried out in NODAT populations following other forms of organ transplant (such as liver transplant) were not included. PubMed, Web of Science and Google Scholar were searched from their inception until May 2015 with no language restrictions, using the following keywords: ‘New Onset Diabetes’, ‘Diabetes Mellitus’, ‘Gene’, ‘Genetic’, ‘Genotype’, ‘Transplantation’, ‘Transplant’, ‘Polymorphism’, ‘Mutation’, ‘NODAT’ and ‘PTDM’ (Post-Transplantation Diabetes Mellitus). Bibliographies for all identified articles and reviews were examined to identify further publications not found in the original search.

### Inclusion Criteria

Studies were included when there was a minimum of three studies investigating the association of a specific variant with NODAT. Studies were deemed eligible if they fulfilled the following criteria: (a) published in a peer reviewed journal article or conference abstract using original data; (b) were conducted in a kidney transplant population in a case-control manner for NODAT; (c) included patients diagnosed with NODAT; (d) included controls who had undergone kidney transplantation but did not develop NODAT during follow-up. Authors were contacted if further essential information was required or if there was a query regarding eligibility. If sufficient information could not be obtained, the study was excluded, as were studies that duplicated data.

### Statistical Analysis

Data was manually extracted from the studies. Information was gathered on study size, numbers of cases versus controls, ethnicity, genotyping methods, recorded odds ratios and p values. If ethnicity was not explicitly stated, this was inferred from the geographical location of the recruitment site and/or contact with authors. Deviation from Hardy-Weinberg Equilibrium was measured using genotype counts with a threshold of p <0.0001. Funnel plots of standard error of the log-odds-ratio against the log-odds-ratio were produced to estimate publication bias. Power calculations were conducted using StatCalc version 6.

Heterogeneity was calculated using a Cochrane Q test for heterogeneity with the I^2^ statistic used to describe percentage variation across studies. Meta-analysis was performed using a random effects model for variants replicated in three or more eligible studies, with significance value set at p <0.05.

All meta-analyses were performed using Review Manager software version 5.3.5 (RevMan 5.3) (http://tech.cochrane.org/revman)[15].

Funnel plots of standard error of the log-odds-ratio against the log-odds-ratio were produced to estimate publication bias. These were assessed by visual inspection. Funnel plots are capable of detecting publication bias which would be undetected by more formal statistical tests. Statistical tests such as Egger’s test were not conducted in this review due to the small number of studies in the meta-analyses, which were not sufficient to distinguish chance from asymmetry.

## Results

### Included Studies

**Fig 1:**
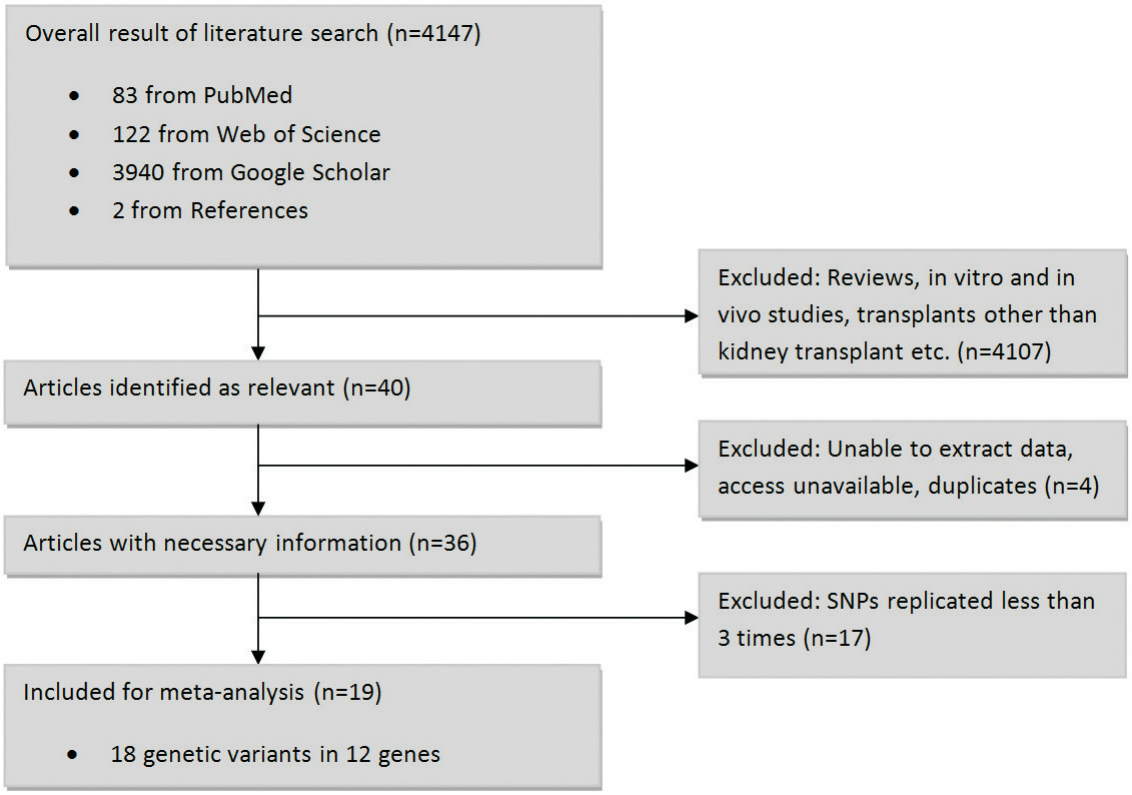
Flowchart describing the process of selection of eligible articles and variants for inclusion in the meta-analysis.

The preliminary literature search yielded 4,147 citations, 40 of which were relevant studies investigating NODAT and 36 of which had all the required information to allow the extraction of variant information (Fig 1). Data was extracted from these articles for all investigated SNPs.

Of the 36 studies deemed eligible for inclusion, 16 studies were carried out in Asian populations, 16 in Caucasian populations and 4 in populations of mixed ethnicity. Table 1 outlines the characteristics of each of the eligible studies.

**Table 1:** Summary of eligible studies describing the ethnicity, genotyping method and total numbers of cases (NODAT patients) and controls (non-NODAT kidney transplant recipients).

Paper	Ethnicity	Genotyping Method	NODAT Cases	Controls	Incidence	NODAT Definition
**Babel 2004** [16]	Caucasian	PCR-SSP	57	221	21%	Based on laboratory tests including FBG ≥126mg/dL
**Cattaneo 2009** [17]	Caucasian	dHPLC & Direct Sequencing	24	123	16%	PGC ≥200mg/dL with symptoms or FBG ≥126mg/dL
**Chakkera 2012** [18]	Mixed	Sequenom iPLEX	22	69	24%	Anti-diabetic medication requirement after one month post-transplant
**Chang 2011** [19]	Asian	PCR-RFLP	81	295	22%	FBG ≥126mg/dL or PGC ≥200mg/dL with symptoms or two hour PGC ≥200mg/dl during OGTT
**Chen 2012** [20]	Asian	TaqMan & RT-PCR	162	157	51%	Two or more FBG >126mg/dL or anti-diabetic medication requirement beyond 30 days post-transplant
**Dutkiewicz 2010** [21]	Caucasian	PCR-RFLP	21	138	13%	HbA1c continuously >6.5%, FBG ≥126mg/dL, or anti-diabetic medication requirement beyond three months post-transplant
**Elens 2013** [22]	Caucasian	RT-PCR	9	76	11%	Use of anti-diabetic medication at any time during the follow up period
**Ergun 2011** [23]	Caucasian	PCR-RFLP	9	73	11%	Symptoms of diabetes with PGC ≥200mg/dL, or two or more consecutive FBG >126mg/dL or two hour PGC ≥200mg/dL during OGTT
**Fougeray 2011** [24]	Mixed	TaqMan	14	255	5%	FBG >126mg/dL or non-fasting glycaemia >11mmol/L measured at baseline or at days 14, 30, 60, 90
**Ghisdal 2009** [25]	Caucasian	RT-PCR	118	958	11%	FBG ≥126mg/dL on two or more occasions or *de novo* anti-diabetic medication requirement
**Jeong 2010** [26]	Asian	Direct Sequencing (ABI-PRISM)	56	255	18%	FPG >126g/dL, HbA1c >6.5 or insulin and oral hypoglycaemic agents required for over 3 months
**Kang 2008** [27]	Asian	TaqMan	174	450	28%	Three months post-transplant began anti-diabetic medication and continued thereafter
**Kang 2008a** [28]	Asian	TaqMan	119	392	23%	One year post-transplant began anti-diabetic medication after continued thereafter
**Kang 2009** [29]	Asian	RT-PCR	145	444	25%	One year post-transplant began anti-diabetic medication after continued thereafter
**Kang 2012** [30]	Asian	TaqMan	154	421	27%	One year post-transplant began anti-diabetic medication after continued thereafter
**Kao 2010** [31]	Asian	PCR-RFLP	73	241	23%	Patients with HbA1c >6.5mg/dL on sequential blood samples
**Khan 2015** [32]	Asian	PCR-RFLP	42	98	30%	Administered anti-diabetic medication for more than three months post-transplant
**Kim 2012** [33]	Asian	Direct Sequencing (ABI-PRISM)	53	253	17%	FBG concentration over 125mg/dL, HbA1c more than 6.5% or anti-diabetic medication required for over 3 months
**Kurzawski 2010** [34]	Caucasian	PCR method	56	158	26%	HbA1c >6.5mg/dL, FBG >126mg/dL or those requiring anti-diabetic medication for greater than three months at one year post-transplant
**Kurzawski 2011** [35]	Caucasian	RT-PCR	66	168	28%	HbA1c >6.5mg/dL, FBG >126mg/dl or those requiring anti-diabetic medication for greater than three months
**Kurzawski 2012** [36]	Caucasian	RT-PCR	67	168	29%	HbA1c >6.5mg/dl, FBG >126mg/dL or those requiring anti-diabetic medication for greater than three months
**Kurzawski 2014** [37]	Caucasian	RT-PCR	48	176	21%	FPG >126mg/dL or those requiring anti-diabetic medication for greater than three months
**Lee 2013** [38]	Asian	Direct Sequencing (ABI-PRISM)	49	253	16%	Three months post-transplant FBG ≥126mg/dL or symptoms of diabetes with PGC ≥200mg/dL at any time of day or two hour PGC ≥200mg/dL during OGTT or anti-diabetic medication required for more than three months
**McCaughan 2014** [1]	Caucasian	Illumina 660K Array & Sequenom iPLEX	26	230	10%	New requirement for anti-diabetic medication after transplant
**Nicoletto 2013** [39]	Caucasian	Sequenom iPLEX RT-PCR	83	187	31%	Second recorded FBG of 126mg/dL or more
**Özdemir 2011** [40]	Caucasian	PCR Method	23	27	46%	Symptoms of diabetes with PGC ≥200mg/dL, or record of two or more consecutive FBG >126mg/dL or two hour PGC ≥200mg/dL during OGTT and anti-diabetic medication requirement
**Szuszkiewicz 2011** [41]	Mixed	PCR-RFLP	36	79	31%	Anti-diabetic medication requirement, FBG ≥126mg/dL and two hour PGC ≥200mg/dL when available from patient history
**Tavira 2011** [42]	Caucasian	PCR-RFLP	115	205	36%	FBG>126g/dL after three consecutive measurements
**Tavira 2012** [43]	Caucasian	PCR-RFLP	115	205	36%	FBG>126g/dL after three consecutive measurements
**Tsai 2011** [44]	Asian	PCR-RFLP	85	198	30%	FBG ≥126mg/dL or symptoms of diabetes and PGC ≥200mg/dL at any time of day or two hour PGC ≥200mg/dL during OGTT
**Vattam 2013** [45]	Asian	PCR-RFLP	42	98	30%	PGC ≥200mg/dL with diabetes symptoms or FBG ≥126mg/dL or two hour PGC ≥200mg/dL during OGTT
**Wang 2011** [46]	Mixed	Direct Sequencing	51	72	41%	FBG ≥126mg/dL three months post-transplant
**Weng 2012** [47]	Asian	PCR-RFLP	27	251	10%	PGC ≥200mg/dL with diabetes symptoms or FBG ≥126mg/dL or two hour PGC ≥200mg/dL during OGTT
**Yang 2011** [48]	Caucasian	RT-PCR	133	170	44%	Two or more occasions of FPG level >126mg/dL one month or more after transplant
**Yao 2013** [49]	Asian	PCR-RFLP	16	89	15%	PGC ≥200mg/dL with diabetes symptoms or FBG ≥126mg/dL or two hour PGC ≥200mg/dL during OGTT
**Yu 2011** [50]	Asian	PCR-RFLP	97	301	24%	FBG ≥126mg/dL on at least two occasions or to require anti-diabetic medication

PCR-SSP, Polymerase chain reaction, single specific primer; dHPLC, Denaturing high performance liquid chromatography; RFLP, PCR Restriction Fragment Length Polymorphism; RT-PCR, Real Time PCR; FBG, Fasting Blood Glucose; PGC, Plasma Glucose Concentration; OGTT, Oral Glucose Tolerance Test; HbA1c, Haemoglobin A1c

**Fig 2:**
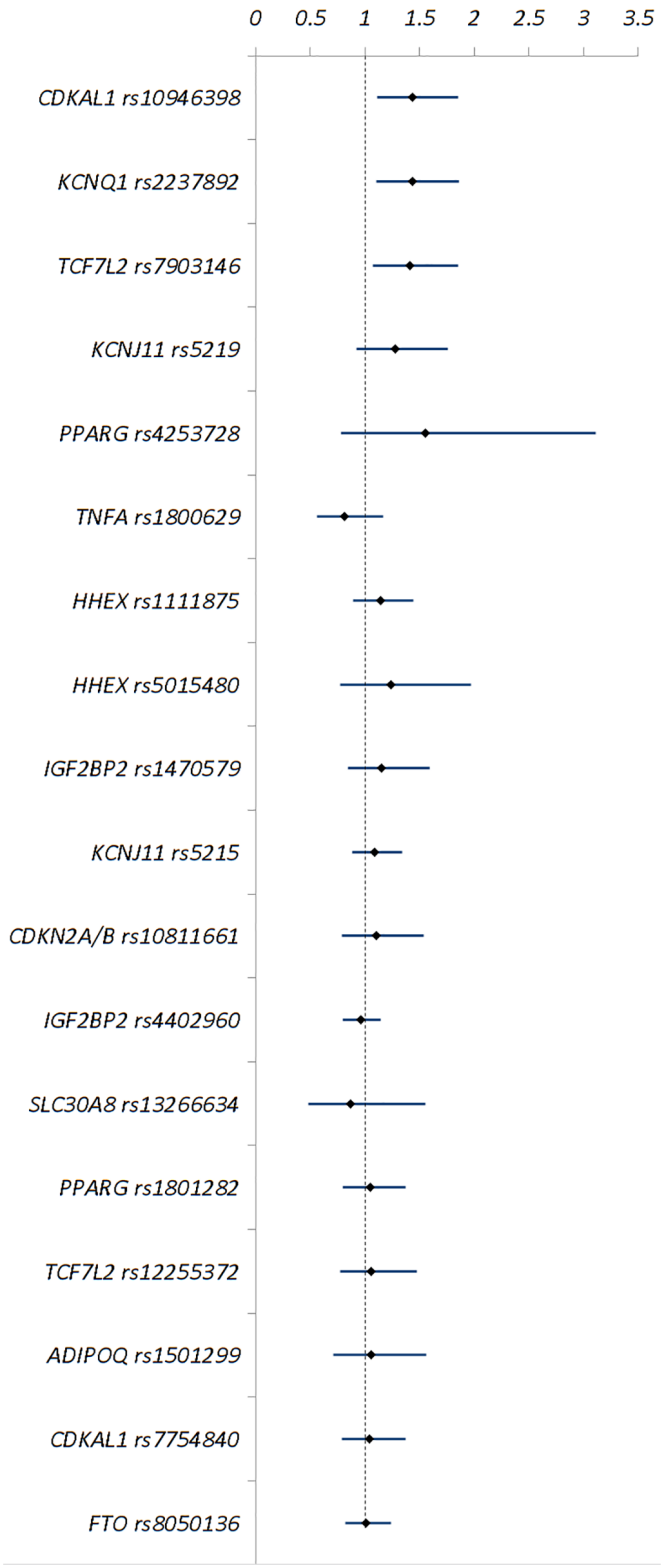
Genetic variants explored for association with NODAT in at least three publications.

The literature review revealed 18 genetic variants considered for association with NODAT that were reported in a minimum of three studies across 12 genes (Table 2 and Fig 2).

**Table 2:** Variants replicated in a minimum of three publications with associated odds ratios, 95% confidence intervals and p values following meta-analysis.

Gene	Variant	Odds Ratio	95% CI	p Value	Minor Allele	Minor Allele Frequency (Control Group)
***CDKAL1***	**rs10946398**	**1.43**	**1.11–1.85**	**0.006**	**C**	**42.65%**
***KCNQ1***	**rs2237892**	**1.43**	**1.10–1.86**	**0.007**	**T**	**43.39%**
***TCF7L2***	**rs7903146**	**1.41**	**1.07–1.85**	**0.01**	**T**	**18.41%**
***KCNJ11***	rs5219	1.28	0.92**–**1.76	0.14	T	35.50%
***PPARG***	rs4253728	1.55	0.78**–**3.11	0.21	A	23.71%
***TNFA***	rs1800629	0.81	0.56**–**1.17	0.25	A	14.07%
***HHEX***	rs1111875	1.14	0.89**–**1.44	0.30	C	49.92%
***HHEX***	rs5015480	1.24	0.77**–**1.97	0.38	C	33.70%
***IGF2BP2***	rs1470579	1.15	0.84**–**1.59	0.39	C	33.26%
***KCNJ11***	rs5215	1.09	0.88**–**1.34	0.42	C	36.07%
***CDKN2A/B***	rs10811661	1.1	0.79**–**1.54	0.57	C	25.87%
***IGF2BP2***	rs4402960	0.96	0.80**–**1.14	0.61	T	31.85%
***SLC30A8***	rs13266634	0.87	0.48**–**1.55	0.63	T	35.99%
***PPARG***	rs1801282	1.05	0.80**–**1.37	0.73	G	10.71%
***TCF7L2***	rs12255372	1.06	0.77**–**1.47	0.73	T	24.11%
***ADIPOQ***	rs1501299	1.06	0.71**–**1.56	0.79	T	8.33%
***CDKAL1***	rs7754840	1.04	0.79**–**1.37	0.80	C	31.50%
***FTO***	rs8050136	1.01	0.82**–**1.24	0.95	A	32.28%

Variants highlighted in bold are those which reached significance at the 5% level.

**Fig 3:**
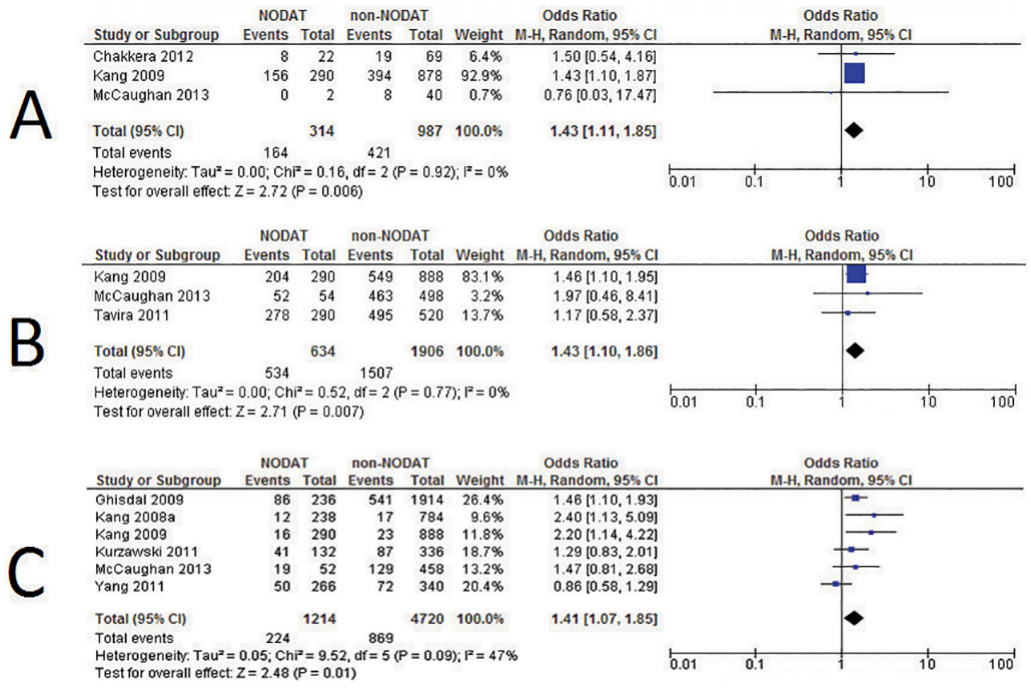
Forest Plots illustrating odds ratios and 95% confidence intervals for the three variants significantly associated with NODAT in random effects meta-analysis; black diamonds represent overall odds ratios for each of the variants. A *CDKAL1* rs10946398 B *KCNQ1* rs2237892 C *TCF7L2* rs7903146.

Of these analysed variants, three were significantly associated with NODAT based on meta-analysis; *CDKAL1* rs10946398 p = 0.006 OR = 1.43, 95% CI = 1.11–1.85 (n = 696 individuals), *KCNQ1* rs2237892 p = 0.007 OR = 1.43, 95% CI = 1.10–1.86 (n = 1,270 individuals), and *TCF7L2* rs7903146 p = 0.01 OR = 1.41, 95% CI = 1.07–1.85 (n = 2,967 individuals) (Fig 3).

Power calculations (S1 Table) suggest this study is adequately powered to identify a risk variant; for example, considering 2360 cases and 607 controls there was >80% power to identify a risk variant with odds ratio 1.5 and minor allele frequency of 5%.

## Discussion

### Main Findings

Thorough investigation of genetic variants reportedly associated with NODAT in kidney transplant patients has revealed significant associations by combined analysis. Three SNPs were significantly associated with NODAT–*TCF7L2* rs7903146, *CDKAL1* rs10946398 and *KCNQ1* rs2237892 at the significance level p<0.05.

Many of the studies used in this investigation focused on genes previously associated with type 2 diabetes (T2D)[29,36]. NODAT and T2D have a number of important similarities. Both are characterised by insulin resistance and insulin hypo-secretion and share similar risk factors including increased age, family history of diabetes and non-white ethnicity [17].

*TCF7L2* (transcription factor 7-like-2) has been previously linked to T2D, and has been cited as one of the most important signals associated with T2D [51]. The T allele was identified as a diabetes risk factor in the pre-GWAS era and was later replicated across a number of groups with different ethnic ancestry [52,53]. It is not yet completely understood how *TCF7L2* influences risk of T2D but a number of theories have been put forward. It may affect blood glucose homeostasis by altering levels of glucagon-like peptide 1 in the gut, or it may decrease insulin secretion *via* the pancreatic beta, adipose or liver cells [54]. rs7903146 is located in an intron; a non-protein coding region of the gene [55]. There is no obvious mechanism by which a mutation at this locus could affect NODAT or T2D development, however the variant rs7903146 may either be in linkage disequilibrium with a causal allele or may itself influence gene expression through regulatory mechanisms.

*CDKAL1* (cyclin dependent kinase 5 regulatory subunit associated protein 1 like 1) has been associated with impaired insulin secretion and the development of T2D in both European and Han Chinese populations by GWAS [56] and the variant rs10946398 has been found to be significantly associated with T2D by meta-analysis [57]. *CDKAL1* encodes a methylthiotransferase which is thought to regulate the CDK5 protein which stimulates production of insulin as well as other processes in the pancreatic beta cells [58]. In this manner, by impairing insulin production via over-expression of CDK5, *CDKAL1* may increase risk of T2D [57] and NODAT. The rs10946398 variant is found in exon 5 of the *CDKAL1* gene. An alternative splicing product of *CDKAL1* (CDKAL1v1) is increased in individuals homozygous for the minor C allele at this locus. It has therefore been suggested that this particular variant influences splicing of the gene [59].

*KCNQ1* is also an established T2D risk factor and has been associated with gestational diabetes [60–62]. Variants of *KCNQ1* cause a variety of disorders including hereditary long QT syndrome (Romano-Ward syndrome)[63]. It is expressed in the pancreatic islet cells as well as the heart and encodes a protein which combines with KCNE proteins to form voltage charged potassium channels found in the membranes. The KCNQ1 proteins form the structure of the channel while the KCNE proteins regulate the activity of the channel [64]. Pancreatic beta cell survival rate is thought to be affected by these potassium channels. It is thought that dysfunction of these potassium channels could alter cell membrane potential and contribute to development of T2D or NODAT. A specific KCNQ1 blocker 293B has been shown to increase insulin production [65]. The variant rs2237892 C risk allele has been shown to be associated with fasting plasma glucose concentration, suggesting that C homozygous individuals have impaired baseline insulin secretion. The gene is also under the control of tissue specific imprinting [66].

These genetic variants are all established T2D risk factors and several variants have been implicated in potential mechanisms contributing to diabetes. Therefore, it is not surprising that these variants are linked to NODAT, another form of diabetes, since the mechanisms controlling insulin production and maintenance of stable glucose levels will both be similar in T2D and NODAT. The meta-analyses conducted on the other variants identified in the literature did not reach statistical significance. This may have been for several reasons, including the small number of studies, small numbers of study participants, or varying phenotypic definitions. Of note, our meta-analysis incorporates data from both candidate gene and genome-wide association studies.

A number of variants which were associated with NODAT in previous studies were not found to be associated with NODAT following meta-analysis. Notable variants which did not reach significance after meta-analysis include *KCNJ11* rs5219 and *ADIPOQ* rs1501299. *KCNJ11* rs5219 is an established T2D risk factor in a gene encoding a voltage gated potassium channel. *ADIPOQ* rs1501299 has been previously associated with NODAT as well as breast cancer, prostate cancer and T2D complications such as heart disease. Adiponectin encoded by *ADIPOQ* is involved in lipid metabolism and insulin sensitivity and making this an attractive candidate for association with NODAT. Neither of these particular variants reached the p<0.05 significance threshold following meta-analysis which could mean they are not associated with NODAT or that the association is only present in certain populations.

### Limitations of the Review

This study does have a number of limitations. The definition of NODAT differs from centre to centre as highlighted by the varying prevalence of NODAT reported in Table 1 which ranges from 5–51%. A potential explanation for this large variation in reported prevalence is the differences in how NODAT is diagnosed i.e. heterogeneity of the clinical phenotype. Some authors employed diagnostic criteria for diabetes as defined by the World Health Organisation and American Diabetic Association, although the final interpretation of these standards did vary in published studies of NODAT. Others authors used pragmatic clinical criteria for NODAT diagnosis defining the affected patients as requiring the *de novo* prolonged use of insulin and/or oral hypoglycaemic medication following transplant. A more rigorously defined NODAT phenotype may facilitate more reproducible results between studies. There were a small number of studies available for many of the variants; larger, carefully phenotyped studies would provide better power to identify alleles robustly associated with NODAT. There was a varying degree of heterogeneity noted between studies, some of which was likely due to different ethnicities considered. In addition, the variations in prescribed immunosuppressive regimens, and their differential effects on NODAT incidence, were not accounted for in many of the studies. It is of note that the Belfast derived data was from the single transplant centre for Northern Ireland. *TCF7L2* rs7903146 variant was only nominally associated with NODAT (p = 0.01), but this association was replicated with the same direction of effect across five independent collections. Possible interactions among the genetic variants identified have not been investigated and this is a further limitation of the study.

## Conclusions

This is a thorough overview of all reported genetic factors influencing the development of NODAT in the current literature. Analysis revealed a significant association between NODAT and three established T2D risk factor variants. Functional studies will be required to further investigate these variants and associated pathways to gain a complete perspective on their effects. In order to obtain more consistency between studies and identify risk alleles with smaller effect sizes, larger participant numbers through multi-centre collaboration and harmonised phenotypic definition of NODAT is required. An adequately powered, dense genome-wide association study will provide more information using a carefully defined NODAT phenotype.

Hypothesis free approaches such as the GWAS carried out by McCaughan and colleagues for NODAT are advantageous to identify new biological targets and therapeutic pathways and should also be carried out in other populations and ethnicities to better understand the genetic architecture underlying this disease [1].

## Supporting Information

S1 Table Power Calculations for Genetic Variants. This table describes the power which this study had to identify significant genetic variants. The power was based on 607 cases, 2360 controls and dependent on the Minor Allele Frequency, significance sought, and effect size (reported for each variant in Table 2).
(XLSX)

S2 Table PRISMA Checklist. Describes how this manuscript conforms to the PRISMA 2009 guidelines.
(DOCX)
